# Accelerated aging in mice with astrocytic redox imbalance as a consequence of SOD2 deletion

**DOI:** 10.1111/acel.13911

**Published:** 2023-08-23

**Authors:** Konstantinos Tsesmelis, Gandhari Maity‐Kumar, Dana Croner, Jasmin Sprissler, Miltiadis Tsesmelis, Tabea Hein, Bernd Baumann, Thomas Wirth

**Affiliations:** ^1^ Institute of Physiological Chemistry University of Ulm Ulm Germany; ^2^ Institute for Diabetes and Obesity Helmholtz Diabetes Center at Helmholtz Zentrum München Neuherberg Germany; ^3^ Department of Pediatrics and Adolescent Medicine Ulm University Medical Center Ulm Germany

**Keywords:** aging, astrocytes, brain, CNS, motoric impairment, oxidative stress, premature aging, SOD2

## Abstract

Aging of the central nervous system (CNS) leads to motoric and cognitive decline and increases the probability for neurodegenerative disease development. Astrocytes fulfill central homeostatic functions in the CNS including regulation of immune responses and metabolic support of neurons and oligodendrocytes. In this study, we investigated the effect of redox imbalance in astrocytes by using a conditional astrocyte‐specific SOD2‐deficient mouse model (SOD2^ako^) and analyzed these animals at different stages of their life. SOD2^ako^ mice did not exhibit any overt phenotype within the first postnatal weeks. However, already as young adults, they displayed progressive motoric impairments. Moreover, as these mice grew older, they exhibited signs of a progeroid phenotype and early death. Histological analysis in moribund SOD2^ako^ mice revealed the presence of age‐related brain alterations, neuroinflammation, neuronal damage and myelin impairment in brain and spinal cord. Additionally, transcriptome analysis of primary astrocytes revealed that SOD2 deletion triggered a hypometabolic state and promoted polarization toward A1‐neurotoxic status, possibly underlying the neuronal and myelin deficits. Conclusively, our study identifies maintenance of ROS homeostasis in astrocytes as a critical prerequisite for physiological CNS aging.

## Graphical abstract

In this study, the antioxidant gene SOD2 was deleted from the astrocytes of mice and the mice were examined during different time points of their life. These mice developed a progeroid phenotype characterized by motoric impairment and early death. Mechanistically, SOD2 deletion in astrocytes triggered a hypometabolic state and polarization toward the highly inflammatory A1 neurotoxic phenotype, hindering their homeostatic functions and leading to multiple aging‐associated brain alterations in a non‐cell‐autonomous way.

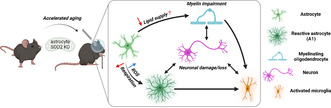

**Abbreviations**
ANOVA: analysis of variance
ATP: adenosine 5′‐triphosphate
BW: body weight
CNS: central nervous system
DMEM: Dulbecco's modified eagle's media
FACS: fluorescence‐activated cell sorting
FDR: false discovery rate
FF: free floating
GO:BP: gene ontology: biological process
GSEA: gene set enrichment analysis
KO: knockout
mROS: mitochondrial reactive oxygen species
NES: normalized enrichment score
OxPhos: oxidative phosphorylation
Par: paraffin
PBS: phosphate‐buffered saline
PCA: principal component analysis
PCR: polymerase chain reaction
PFA: paraformaldehyde
PPP: pentose phosphate pathway
RIPA: radioimmunoprecipitation assay
ROS: reactive oxygen species
SD: standard deviation
SDS: sodium dodecyl sulfate
SNc: substantia nigra pars compacta
SNr: substantia nigra pars reticulata
SOD2ako: SOD2 astrocyte knockout
TBI: traumatic brain injury
TEM: transmission electron microscope
VTA: ventral tegmental area
WT: wild type

## 1 INTRODUCTION

Aging is a condition characterized by progressive accumulation of deleterious changes in cells with advancing age. This leads to cellular dysfunction and corresponding loss of tissue homeostasis, ultimately increasing the risk of disease and death (Harman, 1994). Several theories have been proposed as an explanation for the physiological aging process (Harman, 1998). A well‐accepted theory is the free radical theory of aging proposed by Denham Harman. According to that, the homeostasis of free radical generation and scavenging is disturbed with advanced age. As a consequence, there is accumulation of free radicals which cause a variety of deleterious changes to biomolecules including DNA, protein, and lipid damage (Harman, 1994). These perturbations can induce cellular senescence, a state where the cell is still metabolically active but locked in growth arrest (Courtois‐Cox et al., 2008). Accumulation of damaged and senescent cells in tissue results in a progressive functional deterioration of the corresponding organ and ultimately organismic aging. Commonly observed implications from aging may vary from cognitive decline and memory deficits to motoric impairment, all of which can hinder the everyday life of the aged individual differently.

Mitochondria are the major source of reactive oxygen species (ROS), which are generated as a byproduct of oxidative phosphorylation (OxPhos), and hence tightly connected with the aging process (Jastroch et al., 2010). Therefore, to counter ROS production, mitochondria are well‐equipped with antioxidant enzymes. Superoxide dismutase 2 (SOD2) is a first‐line scavenging enzyme localized in the mitochondrial matrix. SOD2 converts the highly reactive superoxide radical anion (O2^·−^) to the less reactive hydrogen peroxide (H_2_O_2_), thus having a pivotal role in the antioxidant system (Wang et al., 2018). Moreover, SOD2 has been associated with longevity in eukaryotic organisms (Sun et al., 2002) while knockout (KO) studies in mice highlighted its significant role in survival (Lebovitz et al., 1996; Li et al., 1995).

Importantly, malfunction of the antioxidant system leads to an imbalance between the production and inactivation of ROS, a condition called oxidative stress. Oxidative stress has detrimental effects on multiple biological processes and is associated with diverse age‐related and neurodegenerative disorders (Valko et al., 2007). The brain is highly susceptible to oxidative stress since it is one of the most energy‐demanding organs, consuming 20% of the total oxygen intake while only accounting for 2% of the total body mass (Dawson, 1999). Two of the most prevalent neurodegenerative diseases, Alzheimer's and Parkinson's disease, have been associated with increased levels of oxidative stress while the exact cause has not yet been identified so far (Mulica et al., 2021).

Previously, we investigated the role of oxidative stress in principal neurons of the central nervous system (CNS) using a neuron‐specific (CamKIIa‐iCre) SOD2‐deficient mouse model. These mice lacking SOD2 function in neurons die around 4 weeks after birth while developing neuroinflammation, astrogliosis and metabolic alterations (Maity‐Kumar et al., 2015). A slightly more severe phenotype was observed in a brain‐specific (Nestin‐cre) SOD2 deletion study in which SOD2‐deficient mice developed spongiform neurodegeneration, astrogliosis and exhibited shorter survival (Izuo et al., 2015). The presence of neurodegeneration exclusively in the brain‐specific SOD2 KO mice suggests an added effect induced by SOD2 KO in astrocytes or oligodendrocytes. Thus, we aimed to examine the specific role of SOD2 in astrocytes.

Astrocytes are the most abundant glial cell type of the brain (Colombo & Farina, 2016) and they have a broad spectrum of functions. They crosstalk with microglia to regulate neuroinflammation and have an active role in synaptic signaling (Jha et al., 2019; Pellerin et al., 1998; Pellerin & Magistretti, 1994). Furthermore, astrocytes metabolically support neurons and oligodendrocytes and are crucially involved in the maintenance of CNS homeostasis (Camargo et al., 2017; Magistretti & Pellerin, 1999). Finally, as a response to CNS insults (e.g. stroke, TBI), astrocytes can turn reactive, a state called astrogliosis. Reactive astrocytes are characterized by hypertrophy, increased GFAP levels, and generally by an altered gene expression profile (Sofroniew & Vinters, 2010). These alterations strongly vary on the type and the severity of the insult. Based on the alterations in their gene expression, reactive astrocytes were recently classified into either an A1 or A2 phenotype. A2 polarization was originally observed under hypoxic conditions and is considered to be neuroprotective, promoting neuronal survival and tissue repair. On the contrary, A1 polarization was observed after lipopolysaccharide treatment and is considered to exert a detrimental effect on neurons and oligodendrocytes (Ding et al., 2021; Liddelow et al., 2017).

Aging has stark implications on multiple astrocytic attributes. Studies revealed that the number of A1‐polarized reactive astrocytes increases as a result of physiological aging (Clarke et al., 2018), while aged astrocytes also exhibit deregulated cholesterol metabolism (Boisvert et al., 2018). Moreover, aging is associated with changes in the blood–brain barrier, the secretome and the epigenetic signature of astrocytes (Palmer & Ousman, 2018). These alterations potentially contribute to the age‐related functional decline of the CNS. However, the detailed molecular mechanism needs to be clarified.

There has been a previous investigation with respect to elucidating the physiological role of mitochondrial ROS (mROS) in astrocytes (Vicente‐Gutierrez et al., 2019). Expression of a mitochondrial‐tagged catalase transgene in astrocytes resulted in a reduction of ROS and alterations in brain metabolism and mouse behaviour. The authors concluded that homeostatic levels of ROS are required for normal physiology.

Here, we addressed the specific contribution of SOD2 function in astrocytes on ROS homeostasis and overall CNS aging. For this, we generated a new mouse model, where SOD2 is deleted specifically in astrocytes. Ablation of SOD2 in astrocytes did not result in any overt differences in early life. However, when mice grew older, they exhibited a progeroid syndrome and displayed a striking shortening of their lifespan.

## 2 RESULTS

### 2.1 SOD2^ako^ mice show growth retardation and signs of accelerated aging

To investigate the consequences of redox imbalance within astrocytes, we established a mouse model for astrocyte‐specific inactivation of SOD2. To achieve this, we crossed hGFAP‐Cre mice (Zhuo et al., 2001) with SOD2 floxed mice (Strassburger et al., 2005). SOD2 floxed mice have the 3rd exon of the SOD2 gene flanked by loxP sites, so excision of this 39‐amino‐acids exon renders the assembly of a functional SOD2 impossible. The resulting mice will be referred to in this study as SOD2^ako^.

**FIGURE 1:**
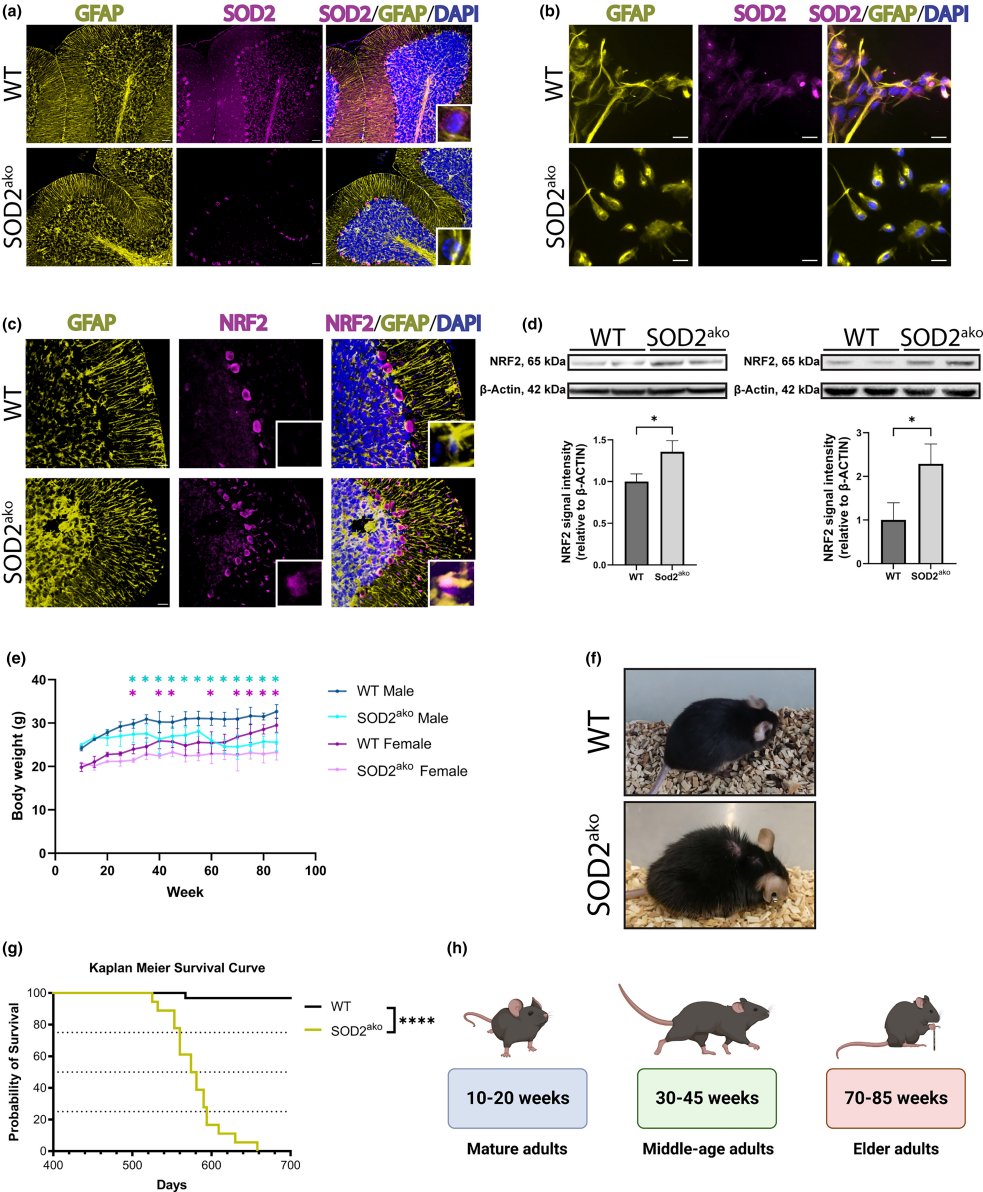
SOD2^ako^ mice show progressive weight loss and reduced lifespan. (a) Validation of the model in vivo. Immunostaining against SOD2 and GFAP at cerebellum of WT and SOD2^ako^ mice. (anti‐SOD2: magenta; anti‐GFAP: yellow, DAPI: blue). Scale bar: 200 μm. (b) Immunostaining of WT and SOD2^ako^ cell cultures against SOD2 and GFAP (anti‐SOD2: magenta; anti‐GFAP: yellow, DAPI: blue). Scale bar: 20 μm. (c) Immunostaining against NRF2 and GFAP at cerebellum of WT and SOD2^ako^ mice. (anti‐NRF2: magenta; anti‐GFAP: yellow, DAPI: blue). Scale bar: 200 μm. (d) Western blot analysis of cerebral (Left) and spinal cord (Right) protein lysates against NRF2. β‐Actin was used as a loading control. Data shown as mean ± SD; *n* = 3 mice/group; student's *t*‐test: * *p* < 0.05. (e) Comparison of the body weight of WT and SOD2^ako^ mice throughout their lifespan. Data shown as mean ± SD; *n* ≥ 4 mice/group; 2‐way ANOVA with Bonferroni correction and multiple comparisons between the age‐matched groups: *p < 0.05 between males, *p < 0.05 between females. (f) Representative picture of mice at 70 weeks. The SOD2^ako^ mice developed piloerection and kyphosis by that age. (g) Kaplan–Meier survival curve for WT (black line) and SOD2^ako^ (yellow line) mice. *n*_WT_ = 20 mice, *n*_SOD2ako_ = 18 mice; log‐rank test: ****p < 0.0001. (h) Mice were categorized into 3 age groups for analysis with regard to the biological aging of the mutants.

SOD2^ako^ mice were born at the expected Mendelian frequency without any discriminating phenotype or any difference in their survival rate. To verify the specific and efficient deletion of SOD2, genomic DNA, biochemical and immunohistochemical studies were performed. Cerebral, cerebellar and spinal cord genomic DNA PCR analysis of GFAP‐Cre negative/SOD2 floxed mice showed just the expected SOD2 floxed band of 850 bp, while in SOD2^ako^ mice, an additional band of 200 bp was observed as well, indicative of the deletion (Figure S1A). To further validate the model, immunofluorescence studies were performed with brain sections using antibodies against SOD2 and GFAP. We could confirm that the presence of SOD2 was limited mainly to non‐GFAP^+^ cells and only a small fraction of GFAP^+^ cells remained positive stained for SOD2 (Figure 1a). Western blot and immunofluorescence analysis of primary astrocytes further confirmed the absence of SOD2 expression in SOD2^ako^ astrocytes (Figure S1B, Figure 1b).

Given the pivotal role of SOD2 in the antioxidant system, we investigated whether its deletion promotes redox imbalance. Thus, we assessed NRF2 levels, a transcription factor activated by oxidative stress and therefore indicative for the cellular redox status. Specifically, under non‐stressed conditions, NRF2 is bound to KEAP1, anchored in the cytoplasm and subsequently degraded. In contrast, under conditions of oxidative stress, NRF2 detaches from KEAP1. NRF2 is therefore stabilized and free to translocate into the nucleus where it regulates transcription by promoting the expression of genes with the Antioxidant Response Element (ARE) sequence. Immunofluorescence analysis of cerebellar brain slices revealed increased levels of NRF2 in the brains of SOD2^ako^ mice in the molecular and granular layer but also in the Purkinje cell monolayer (Figure 1c). Further, western blot analysis revealed increased levels of NRF2 at the cerebrum and spinal cord of SOD2^ako^ animals (Figure 1d), as well as increased levels of HO‐1, an NRF2 target gene (Figure S1C).

Interestingly, at the age of 20 weeks, SOD2^ako^ mice started to show some growth retardation resulting in significantly lower body weight at the age of 30 weeks (Figure 1e). At 50 weeks of age, SOD2^ako^ mice weighted approximately 10% less than their age‐matched control littermates. As SOD2^ako^ animals grew older, they developed kyphosis, piloerection and resting tremor (Figure 1f) accompanied by impaired gait and mobility. At 75–85 weeks of age, mutants developed hindlimb paresis, marking the humane endpoint of the animals. Most SOD2^ako^ animals got moribund at age of around 85 weeks (Figure 1g). Based on this phenotype development, we categorized the animals for analysis into 3 different age groups with regard to the biological age of the mutants; mature adults (10–20 weeks), middle‐age adults (30–45 weeks) and elder adults (70–85 weeks) (Figure 1h).

### 2.2 SOD2^ako^ mice develop progressive motoric impairment

**FIGURE 2:**
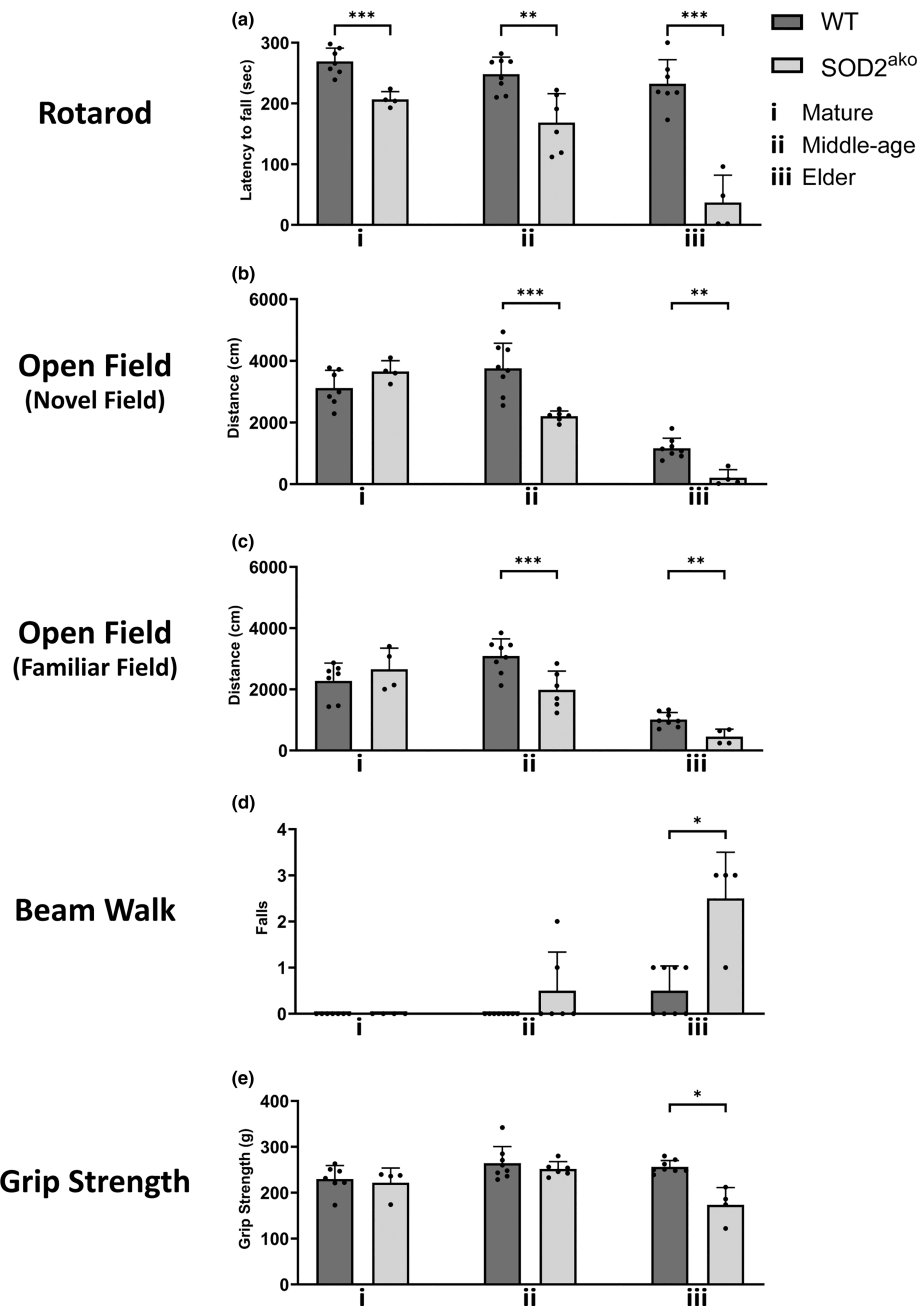
Motor‐behavioral assessment revealed motoric alterations in SOD2^ako^ mice. (a) Latency to fall in a Rotarod test. (b, c) Total distance travelled in (b) a novel and in (c) a familiar field. (d) Failed attempts to successfully cross a beam in a beam walk test. (e) Muscular strength (all limbs) assessed in a grip strength analysis. Data in this figure shown as mean ± SD; *n* = 4–8 mice/group; student's *t*‐test: *p < 0.05, **p < 0.01, ***p < 0.001.

To characterize the behavioural alterations of the SOD2^ako^ mice in detail, we performed a number of tests. Specifically, mice underwent the rotarod and beam walk test to evaluate their balance and motor coordination, the open field test (novel field, familiar field) to assess their activity and exploratory behaviour, and finally, the grip strength test (all limbs) to assess their muscular strength. Although mature SOD2^ako^ mice did not display any obvious motoric deficit, they always performed worse at the rotarod test than their control littermates, staying approximately 25% less time on the rod (Figure 2ai). However, we did not observe any difference in the other tests for the mature age group (Figure 2bi,ci,di,ei).

Analysis of the middle‐age group revealed that aging worsened their performance on the rotarod. Specifically, SOD2^ako^ mice stayed approximately 32% less time on the rod than age‐matched controls (Figure 2aii). Furthermore, open field tests showed that SOD2^ako^ mice exhibited decreased exploratory activity and hypoactivity. We determined significantly less movement during both the novel field and familiar field open field test, approximately 42% and 37% less distance traveled respectively (Figure 2bii,cii). Still, no difference in the beam walk or the grip‐strength test was observed for the middle‐age group (Figure 2dii,eii).

Behavioural analysis of the elder group revealed that the mutant mice's hypoactivity and imbalance on the rod further deteriorated. The elder mutants stayed 85% less time on the rod than their WT littermates (Figure 2aiii), while they also traveled 85% and 56% less distance for the novel field and the explored field, respectively (Figure 2biii,ciii). In addition, 75% of mutants failed to cross the beam in all trials for the beam walk test (Figure 2diii). At that time, they also showed for first time decreased muscular strength as they exhibited 32% decreased force at the grip strength test (Figure 2eiii). Notably, the mutants' observed phenotype was limited to motoric alterations. SOD2 deletion did not lead to any anxiety‐related behaviour in any age according to the corner/center stay ratio during the open field tests (data not shown). It is also worth mentioning that some mature and middle‐age mutants exhibited sporadically seizure‐like psychotic moments (Video S1).

### 2.3 SOD2 deletion in astrocytes induces astrogliosis and microgliosis

To understand the reasons underlying this behavioral phenotype development, we performed macroscopical examination of the brains which did not reveal any overt difference. However, measurement of brain length and width revealed a slight but significant reduction in the size of the elder SOD2^ako^ group (Figure S2A,B). This kind of brain shrinkage is a characteristic observed in physiological aging but also in many neurodegenerative disorders (Pini et al., 2016).

**FIGURE 3:**
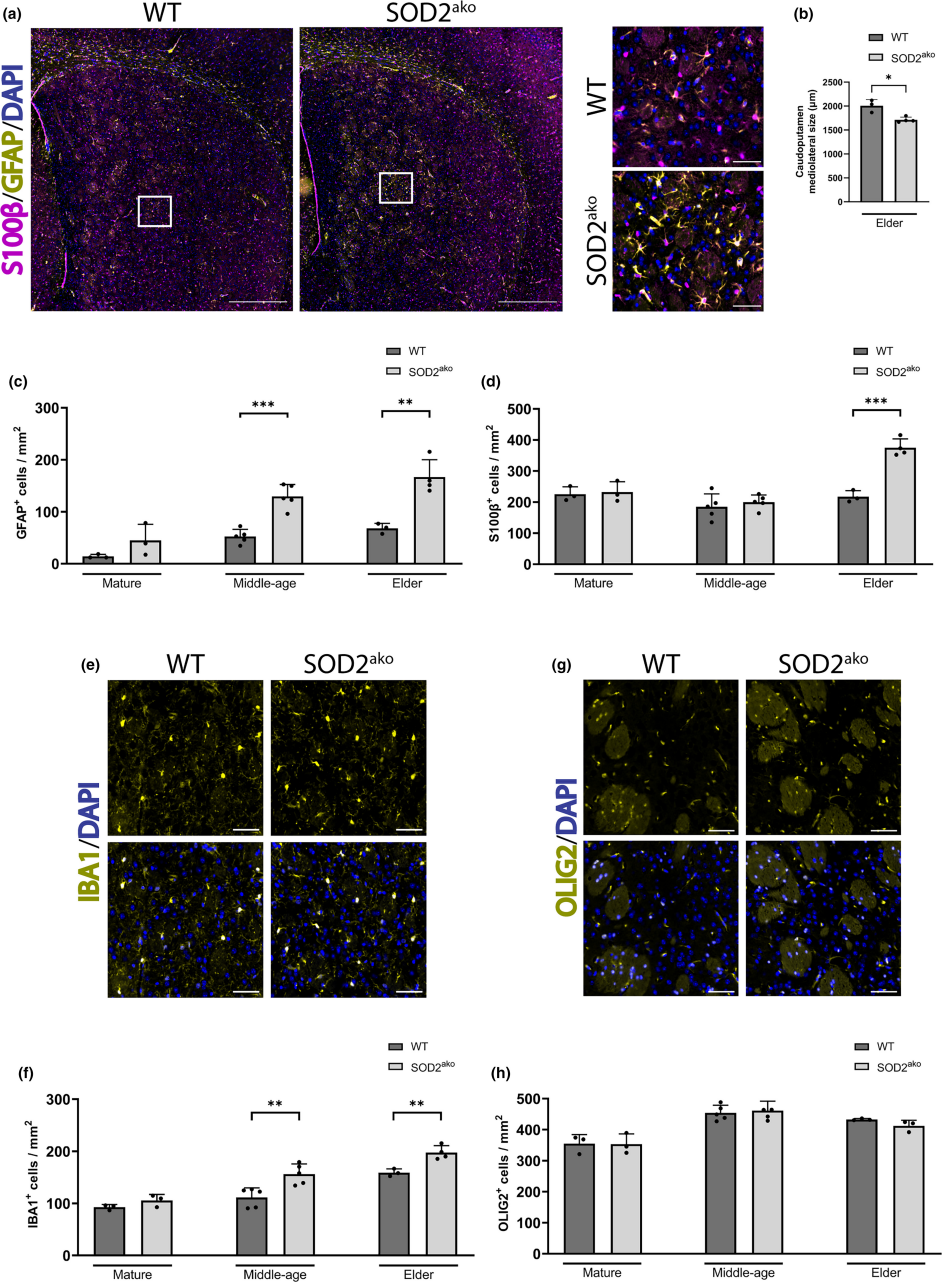
SOD2^ako^ mice exhibit astrogliosis and microgliosis. (a) Immunostaining against GFAP and S100β at the caudoputamen of elder mice (anti‐S100B: magenta; anti‐GFAP: yellow, DAPI: blue). Full image scale bar: 500 μm, inset scale bar: 50 μm. (b) Quantification of caudoputamen mediolateral size between elder WT and SOD2^ako^ mice. (c) Quantification of GFAP^+^ cells at the caudoputamen throughout aging. (d) Quantification of S100β^+^ cells at the caudoputamen throughout aging. (e) Immunostaining against IBA1 at the caudoputamen of elder mice (anti‐IBA1: yellow; DAPI: blue). Scale bar: 50 μm. (f) Quantification of IBA1^+^ cells at the caudoputamen throughout aging. (g) Immunostaining against OLIG2 at the caudoputamen of elder mice (anti‐OLIG2: yellow; DAPI: blue). Scale bar: 50 μm. (h) Quantification of OLIG2^+^ cells at the caudoputamen throughout aging. Data in this figure shown as mean ± SD; *n*_WT‐mature_ = 3 mice, *n*_SOD2ako‐mature_ = 3 mice, *n*_WT‐middle‐age_ = 5 mice, *n*_SOD2ako‐middle‐age_ = 5 mice, *n*_WT‐elder_ = 3 mice, *n*_SOD2ako‐elder_ = 4 mice; student's *t*‐test: *p < 0.05, *p < 0.05, **p < 0.01, ***p < 0.001.

We further analyzed different anatomical brain domains focusing mainly on areas responsible for motor control. Consistent with the overall brain size reduction, elder SOD2^ako^ had a smaller caudoputamen, as indicated by the medial to lateral edge length measurement (Figure 3a,b). We also quantified the number of astrocytes in the same area by S100β and GFAP immunostaining at all three time points. Regarding the mature age group, we observed an increase in the number of reactive astrocytes but only in some SOD2^ako^ mice (Figure 3c,d, Figure S2C). In contrast, in the middle‐age group, all SOD2^ako^ mice had significantly more reactive astrocytes compare to WT mice, while the absolute number of astrocytes was not altered (Figure 3c,d). Finally, in the elder SOD2^ako^ group, we detected an increase in the absolute number of astrocytes as well (Figure 3a,c,d). Similar changes were also observed in the motor cortex, cerebellum and substantia nigra, areas involved in motor regulation (Figure S2D–I). Further, we quantified the surface covered by reactive astrocytes and detected hypertrophic reactive astrocytes starting from the middle‐age group onwards (Figure S2J). These data indicate the development of astrogliosis upon SOD2 deletion.

Under pathologic conditions astrocytes crosstalk with microglia. This interaction can induce an increase in the number and reactivity of microglial cells, a condition termed microgliosis. IBA1 staining revealed increased number of microglia cells which chronologically coincided with the initiation of astrogliosis, starting in the middle‐aged group (Figure 3e,f, Figure S2K,L). Lastly, we also assessed the brain for alterations at the oligodendrocyte linage cells but did not observe any difference in their number at any time point, as indicated by immunofluorescence analysis (Figure 3g,h).

### 2.4 Deletion of SOD2 in astrocytes leads to myelin impairment and neuronal insults

We further investigated the mechanism underlying the observed motoric phenotype. Demyelinating disorders have been tightly correlated with motoric impairment. Moreover, neuronal insults in both higher and lower motor neuron regions can lead to motoric deficits. Thus, we assessed the myelin as well as the brain and spinal cord neuronal integrity.

**FIGURE 4:**
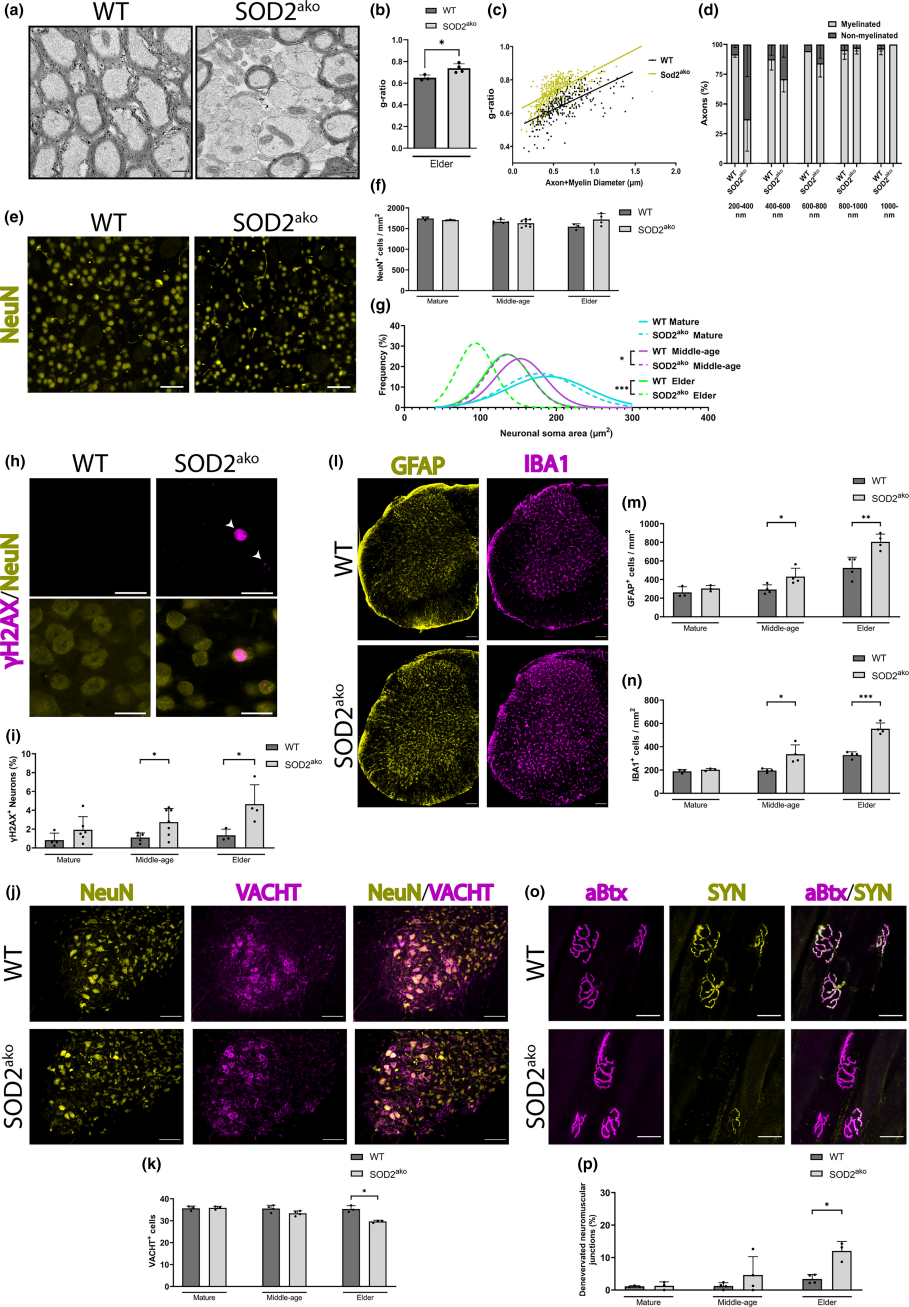
Elder SOD2^ako^ mice show neuronal insults and myelin impairment at the CNS. (a) Representative electron microscopy picture from axon fiber bundles at the caudoputamen of elder mice. Scale bar: 250 nm. (b) Quantification of g‐ratio from axon fiber bundles at the caudoputamen of elder mice. Data shown as mean ± SD; *n*_WT_ = 3 mice, *n*_SOD2ako_ = 4 mice; student's *t*‐test: *p < 0.05. (c) Scatter plot graph displays *g*‐ratio (*y*‐axis) in relation to axon diameter (*x*‐axis) from axon fiber bundles at the caudoputamen of elder mice. *n*_WT_ = 329 axons, *n*_SOD2ako_ = 374 axons. (d) Quantification of myelinated and non‐myelinated axons from the caudoputamen of elder mice (categorized in groups based on their caliber). Data shown as mean ± SD; *n*_WT_ = 3 mice, *n*_SOD2ako_ = 4 mice. (e) Immunostaining against NeuN at the caudoputamen of elder mice (anti‐NeuN: yellow). Scale bar: 50 μm. (f) Quantification of NeuN^+^ cells at the caudoputamen throughout aging. Data shown as mean ± SD; *n*_WT‐mature_ = 3 mice, *n*_SOD2ako‐mature_ = 3 mice, *n*_WT‐middle‐age_ = 5 mice, *n*_SOD2ako‐middle‐age_ = 8 mice, *n*_WT‐elder_ = 3 mice, *n*_SOD2ako‐elder_ = 4 mice; student's *t*‐test (g) Gaussian distribution of neuronal soma size at the caudoputamen throughout aging. *n* = 222–400 neurons/group; student's *t*‐test between age‐matched groups: *p < 0.05, ***p < 0.001. (h) Immunostaining against γH2AX and NeuN at the caudoputamen of elder mice (anti‐γH2AX: magenta, anti‐NeuN: yellow). Scale bar: 20 μm. (i) Quantification of γH2AX^+^/NeuN^+^ cells at the caudoputamen throughout aging. Data shown as mean ± SD; *n*_WT‐mature_ = 4 mice, *n*_SOD2ako‐mature_ = 6 mice, *n*_WT‐middle‐age_ = 5 mice, *n*_SOD2ako‐middle‐age_ = 7 mice, *n*_WT‐elder_ = 3 mice, *n*_SOD2ako‐elder_ = 4 mice; student's *t*‐test: *p < 0.05. (j) Immunostaining against VACHT and NeuN at the ventral horn of the lumbar region of the spinal cord in elder mice (anti‐NeuN: yellow, anti‐VACHT: magenta). Scale bar: 200 μm. (k) Quantification of VACHT^+^ cells at the ventral horn of the lumbar region of the spinal cord throughout aging. Data shown as mean ± SD; *n* = 3–4 mice/group; student's *t*‐test: *p < 0.05. (l) Immunostaining against GFAP and IBA1 at the lumbar region of the spinal cord (anti‐GFAP: yellow, anti‐IBA1: magenta). Scale bar: 100 μm. (m) Quantification of GFAP^+^ cells at the grey matter of the lumbar region of the spinal cord throughout aging. Data shown as mean ± SD; *n* = 3–4 mice/group; student's *t*‐test: *p < 0.05, **p < 0.01. (n) Quantification of IBA1^+^ cells at the gray matter of the lumbar region of the spinal cord throughout aging. Data shown as mean ± SD; *n* = 3–4 mice/group; student's *t*‐test: *p < 0.05, ***p < 0.001. (o) Immunostaining against SYN with the addition of aBTX at the gastrocnemius muscle (a‐Btx: magenta, anti‐SYN: yellow). Scale bar: 30 μm. (p) Quantification of denervated neuromuscular junctions at the gastrocnemius muscle throughout aging. Data shown as mean ± SD; *n* = 3–4 mice/group; student's *t*‐test: *p < 0.05.

While we did not observe any difference in the number of oligodendrocyte lineage cells in any age group, electron microscopy analysis revealed alterations in the myelin composition of the axon fiber bundle at the caudoputamen of elder SOD2^ako^ mice. We also observed an abnormal structure at a fraction of the axon fiber bundles passing through the caudoputamen of the mutant mice, indicating possible axonal loss (Figure S3A). Furthermore, the SOD2^ako^ mutant axons had thinner myelin layers as indicated by g‐ratio analysis (Figure 4a–c), and slightly although non‐significant decreased caliber overall (Figure S3B). Interestingly, we also observed a higher number of non‐myelinated axons in SOD2^ako^ mice (Figure S3C), while further categorization of these axons based on their caliber revealed that the majority of the non‐myelinated axons belonged to the smaller size groups (200–600 nm) (Figure 4d).

Further, we assessed the brain for potential neurotoxic effects, and quantified the number of neurons and evaluated their condition and health. Immunofluorescence studies did not reveal any difference in the number of neurons in the brain between the 2 groups at any age, as visualized by NeuN staining (Figure 4e,f). Of note, we observed a striking decrease in the size of the soma of neurons from the middle‐age group onwards (Figure 4e, Figure S3D). Neuronal shrinkage generally occurs physiologically with aging but can also appear under pathological conditions. Gaussian frequency distribution of the size of neurons revealed that neurons from the middle‐age mutants had a similar distribution to the neurons from the elder WT group (Figure 4e,g), a possible sign of accelerated aging. This observation prompted us to test neuronal integrity. Hence, the accumulation of DNA damage in neurons was examined by γH2AX staining, a marker indicative of DNA damage. Here, we detected an increased number of damaged neurons starting from the middle‐age group onwards in the brains of SOD2^ako^ mice (Figure 4h,i).

Finally, histological analysis at the lumbar region of the spinal cord revealed a loss of motor neurons at the ventral horn of the SOD2^ako^ mice in the elder group, while no difference was observed in the younger age groups (Figure 4j,k). We also observed an increased number of reactive astrocytes and microglia in the gray matter of the spinal cord of SOD2^ako^ mice starting from the middle‐aged group, preceding the loss of motor neurons (Figure 4ln). Additionally, we identified neuromuscular denervation, a characteristic observed in motor‐neuron diseases, such as Amyotrophic Lateral Sclerosis (ALS) and Spinal Muscular Atrophy (SMA). Examination of the gastrocnemius muscle revealed an increased number of denervated neuromuscular junctions in elder SOD2^ako^ mice, as indicated by a‐Bungarotoxin (a‐Btx)/Synaptophysin (SYN) staining, while no difference was observed in the younger groups (Figure 4o,p).

Altogether these data reveal deficits in myelination and neuronal degeneration in both the brain and the spinal cord of SOD2^ako^ mice, potentially explaining the observed motoric phenotype.

### 2.5 SOD2^ako^ astrocytes adopt a hypometabolic state and A1 reactive phenotype

**FIGURE 5:**
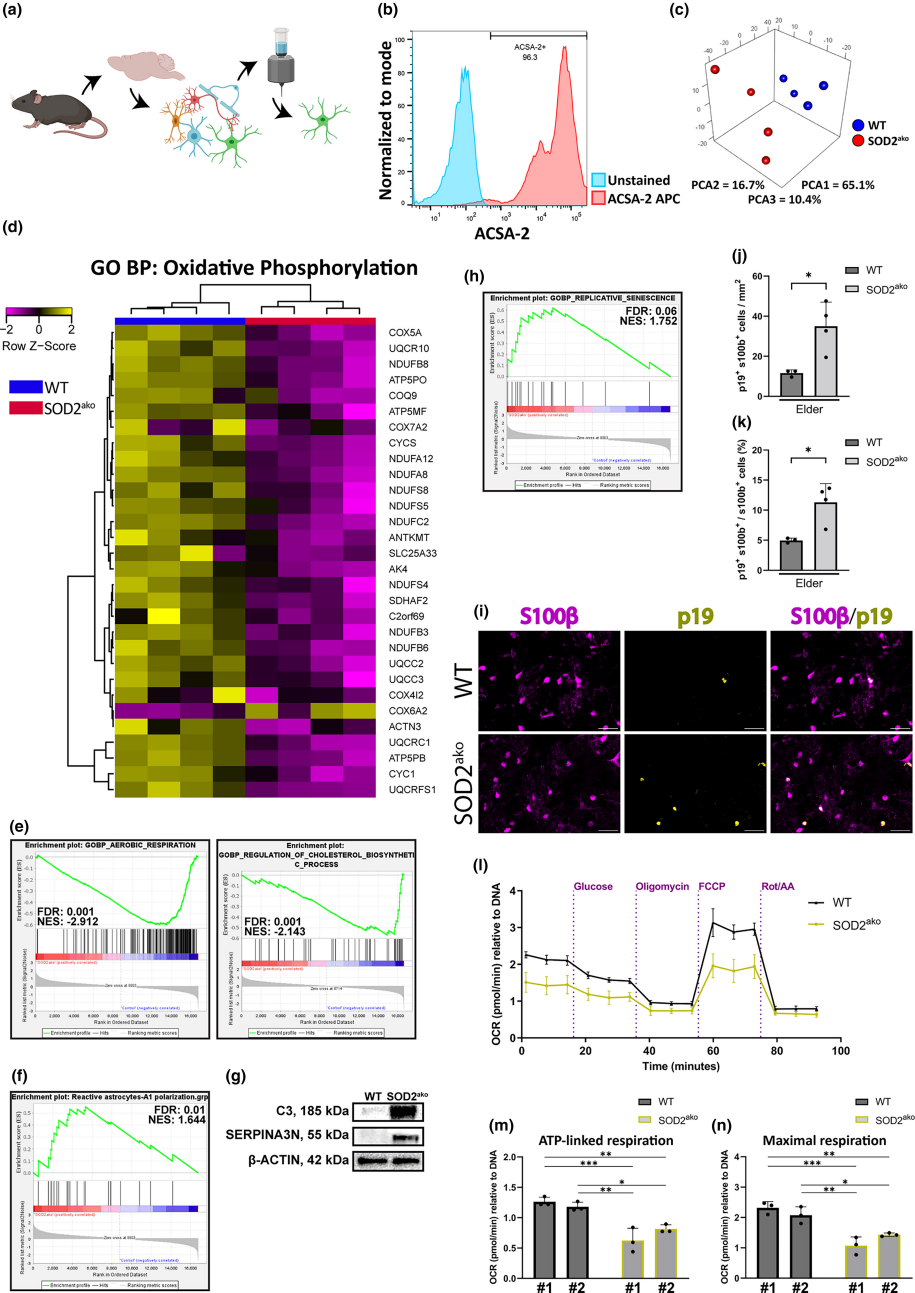
RNAseq analysis revealed metabolic alterations and polarization toward the A1 phenotype in elder SOD2^ako^ astrocytes. (a) Immunomagnetic approach for isolation of astrocytes (ACSA‐2^+^ fraction) from dissociated brain lysate. (b) Representative flow cytometry measurement to validate the efficiency of acutely isolated astrocytes (anti‐ACSA‐2 conjugated to APC). (c) 3D PCA plot of the samples used for RNAseq analysis. (PCA1: 65.1%, PCA2: 16.7%, PCA3: 10.4%). (d) Heatmap of the top 30 regulated genes from GO:BP Oxidative Phosphorylation geneset. *n* = 4 samples/group. (e) GSEA analysis of the aerobic respiration (left) and the cholesterol biosynthetic process (right) from astrocytes of elder mice. *n* = 4 mice/group; GOBP Aerobic respiration: FDR = 0.001, NES = −2.912. GOBP Regulation of cholesterol biosynthetic process: FDR = 0.001, NES = −2.143. (f) GSEA analysis of A1 reactive phenotype from astrocytes of elder mice. *n* = 4 samples/group. Reactive astrocytes – A1 polarization: FDR = 0.01, NES = 1.644. (g) Western blot analysis of acutely isolated astrocyte protein lysates against C3 and SERPINA3N in the brains of SOD2^ako^ mice. β‐Actin was used as a loading control. *p < 0.05, **p < 0.01, ***p < 0.001, ****p < 0.0001. (h) GSEA analysis of replicative senescence from astrocytes of elder mice. *n* = 4 samples/group. Replicative senescence: FDR = 0.06, NES = 1.752. (i) Immunostaining against p19 and S100β at the caudoputamen of elder mice (anti‐S100B: magenta; anti‐p19: yellow, DAPI: blue). Scale bar: 30 μm. (j) Quantification of p19^+^/S100b^+^ cells at the caudoputamen of elder mice. (k) Percentage of p19^+^ astrocytes at the caudoputamen of elder mice. (l) Mitochondrial respiration assessment of astrocytes in vitro from 2 WT and 2 SOD2^ako^ mice through a Seahorse assay. Injections with glucose, oligomycin, FCCP, rotenone/antimycin‐A were used in order to calculate basal, maximal and ATP‐linked respiration and spare capacity. Data shown as mean ± SD; *n* = 6/group. (m, n) Quantification of (m) ATP‐linked and (n) maximal respiration from a Seahorse assay. Data shown as mean ± SD; *n* = 3/group; 1‐way ANOVA with Bonferroni correction and multiple comparisons between the WT and SOD2^ako^ groups: *p < 0.05, **p < 0.01, ***p < 0.001.

To elucidate the involved molecular mechanisms, we isolated primary astrocytes from the elder group using an immunomagnetic approach (Figure 5a). Astrocyte purity (over 95% for every sample) was verified by flow cytometry analysis (Figure 5b), and subsequently, RNAseq analysis was conducted with extracted RNA. 3D PCA revealed that the SOD2^ako^ astrocytes deviated from the control group, indicating a strong change at the transcriptome level upon SOD2 deletion (Figure 5c). To identify relevant differentially regulated functions, we performed Gene Set Enrichment Analysis (GSEA). GSEA showed that most metabolic pathways signatures (oxidative phosphorylation, lipid metabolism, pentose phosphate pathway) were significantly depleted at SOD2^ako^ astrocytes indicating deregulation of the astrocytic metabolism (Figure 5d,e, Figure S4A).

We also observed an upregulation of genes associated with astrogliosis and the polarization of the astrocytes toward the A1 phenotype in our RNAseq data which prompted us to design our own geneset for A1‐polarized reactive astrocytes based on the current literature (Boisvert et al., 2018) (list provided at Section 5). The abovementioned signature was highly enriched in SOD2^ako^ astrocytes compared to WT astrocytes (Figure 5f). In addition, we extracted proteins from immunomagnetically isolated astrocytes from elder mice and performed immunoblotting against complement component 3 (C3) and serine protease inhibitor A3N (SERPINA3N). These proteins are generally reported to be upregulated in A1 reactive astrocytes and were among the top‐regulated genes in our GSEA. Immunoblot studies confirmed our RNAseq data, as we found a strong upregulation of both proteins in the astrocytes of elder SOD2^ako^ mice (Figure 5g).

Considering the highly inflammatory A1 phenotype which is associated with aging (Boisvert et al., 2018) and multiple accelerated‐aging observations in the mutant mice, we assessed the senescence state of the mutant astrocytes. Examination of our RNAseq data revealed an enrichment of the replicative senescence geneset in elder SOD2^ako^ astrocytes (Figure 5h). In addition, we performed an immunostaining against S100β and p19, a well‐established senescence marker. We observed an elevated number of p19^+^ astrocytes in the caudoputamen of elder mice (Figure 5i–k). Taken together, these data indicate that SOD2^ako^ astrocytes present signs of accelerated aging.

Since the RNAseq analysis revealed depleted metabolic signatures, we further investigated the cell metabolism of SOD2^ako^ astrocytes. To do so, we established primary astrocytic cultures using astrocytes magnetically isolated from dissociated brains of mature animals while healthy cultures from older animals could not be established due to increased age. We assessed mitochondrial respiration (Figure S4B) and the glycolytic rate of the astrocytes by Seahorse assay. SOD2^ako^ astrocytes showcased decreased ATP‐linked respiration (ATP production), maximal respiration (Figure 5l–n) and a tendency for reduced glycolytic rate (Figure S4C). Altogether, these results indicate the presence of a hypometabolic state in SOD2^ako^ astrocytes.

## 3 DISCUSSION

Aging remains a poorly understood biological phenomenon. Elevated levels of oxidative stress is one of the most accepted hallmarks for the induction of either physiological or accelerated aging. The CNS is extremely vulnerable to aging since it consumes 20% of the body's oxygen and is highly susceptible to oxidative stress. In the present study, we investigated the impact of astrocytic redox imbalance on CNS function. Astrocyte‐specific deletion of SOD2 does not result in immediate consequences at the organismal level, as SOD2 function seemed to be compensated in early life but gets essential with progressing age. SOD2^ako^ animals developed prominent gliosis, myelin deficits and neuronal damage leading to paresis and moribundity at around 85 weeks of age.

Superoxide anion (O2^·−^), a highly reactive molecule, is generated in the mitochondria as a result of electron leak from the electron transport chain and is scavenged by SOD2 (Chen et al., 2003; Jastroch et al., 2010). Therefore, SOD2 is critical in the antioxidant protection and has been linked with longevity of eukaryotic organisms. Specifically, Sun et al demonstrated that overexpression of SOD2 in *Drosophila melanogaster* increased their life expectancy by up to 37% (Sun et al., 2002). Further, conventional SOD2 KO mice died in their first weeks of life while developing dilated cardiomyopathy, acidosis, neurodegeneration and progressive motor disturbances (Lebovitz et al., 1996; Li et al., 1995). Additional studies with cell‐specific deletion of SOD2 resulted in varying outcomes. For instance, fibroblast‐ or skeletal muscle‐specific SOD2 deletion reduced the overall survival of the mice (Treiber et al., 2011), while hepatocyte‐specific SOD2 deletion did not lead to any overt phenotype (Cyr et al., 2013). Finally, concerning the CNS, brain‐specific (Nestin‐cre) or excitatory neuron‐specific (CamKIIa‐iCre) deletion of SOD2 resulted in neurodegeneration in different brain areas accompanied by gliosis while the animals showed early postnatal lethality (Izuo et al., 2015; Maity‐Kumar et al., 2015).

In contrast, SOD2^ako^ mice were born without any discriminating phenotype or any difference in their survival and developed normally in their first 20 weeks of life. Surprisingly though, we identified slight motoric disturbances by the Rotarod test, indicating the initiation of a motoric phenotype already at the age of 20 weeks. From about 30 weeks on, the mutants presented growth retardation, a characteristic that was also observed in other SOD2 KO studies but at a much younger age (Lebovitz et al., 1996; Maity‐Kumar et al., 2015; Treiber et al., 2011). The motoric disturbances deteriorated over time and the mice exhibited a premature onset of age‐related characteristics such as kyphosis and piloerection at their last stage of life. We hypothesize that the development of astrogliosis, microgliosis and neuronal damage at the caudoputamen of the striatum are strongly correlated with the observed motoric phenotype. Caudoputamen has a major regulatory role in the motoric circuit of the brain. Therefore, insults in that area may have an impact on the motoric attributes of the individual.

While in preparation for the manuscript, a study with conditional SOD2 ablation in astrocytes of around 1‐year‐old mice (aSOD2‐KO) was published (Baier et al., 2022). Interestingly, inactivation of SOD2 at that age did not affect the healthspan of the animals and no difference in their body weight, motoric abilities or lifespan was observed. These data highlight that the age at which SOD2 in astrocytes is deleted greatly affects the outcome and astrocytic redox imbalance at younger age exerts a much stronger effect on the health of the mice. Similar to our findings, astrocytic SOD2 KO in 1‐year‐old animals induced astrogliosis, although no further comment on the polarization of these astrocytes was made. Further, male aSOD2‐KO mice exhibited impaired spatial working memory and reduced hippocampal long‐term potentiation while no difference was observed for the corresponding female mice. These alterations were mainly attributed to reduced D‐serine levels in the hippocampus of these animals. In our study, we did not investigate spatial working memory as we focused on the strong motoric phenotype and the brain areas associated with motoric functions.

A previous brain‐specific SOD2 KO study using the Nestin‐Cre mouse model, targeting the whole neurogenic lineage including neurons, astrocytes and oligodendrocytes, reported the development of spongiform neurodegeneration accompanied by astrogliosis already in the early postnatal life (Izuo et al., 2015). However, CamKIIa‐iCre SOD2 KO mice, where SOD2 deletion takes place only at the excitatory neurons, did not develop any spongiform pathology, concluding that loss of SOD2 in non‐neuronal brain cells was necessary for the development of this phenotype (Maity‐Kumar et al., 2015). Interestingly, SOD2 KO at astrocytes in SOD2^ako^ mice did not lead to spongiform encephalopathy either, suggesting that either that SOD2 loss in oligodendrocytes, or SOD2 loss in more than one neurogenic lineage cell types is essential for the development of spongiform encephalopathy. Nevertheless, SOD2^ako^ mice developed astrogliosis and microgliosis in both brain and spinal cord, but only with advancing age (starting at 30 weeks after birth).

Specifically, we observed more GFAP^+^ astrocytes in the middle‐age group mutants than in the middle‐age WT mice, indicating increased number of reactive astrocytes. In addition, we observed an elevated number of GFAP^+^ and S100β^+^ astrocytes in the elder group mutants compared to the age‐matched WT, suggesting both increased reactivity and proliferation. These data imply the presence of mild astrogliosis at younger ages and its consequent progression toward severe astrogliosis with advancing age, according to the gliosis severity criteria reviewed by Sofroniew (Sofroniew & Vinters, 2010). Furthermore, GSEA revealed polarization of elder SOD2^ako^ reactive astrocytes toward the A1 neurotoxic phenotype, which potentially explains the observed neuronal and myelin impairment. The increased astrogliosis severity with advancing age also coincides chronologically with an elevation in the number of damaged neurons at the caudoputamen of elder SOD2^ako^ animals and could partially explain the motoric deterioration of these animals with aging. Finally, mutant astrocytes reach senescence earlier, most likely as a result of redox imbalance or the higher proliferation rate, possibly contributing to the aforementioned phenotype.

Of note, we discovered that the brains of SOD2^ako^ mice were slightly smaller in size. Brain shrinkage is a naturally occurring phenomenon with aging as a consequence of both white and grey matter loss but is also observed under neuropathologic conditions like in the case of Alzheimer's disease (Pini et al., 2016). In the case of aging, gray matter loss is mainly attributed to shrunk neurons and decreased numbers of neuronal spines and synapses (Thulborn et al., 2016) with the most affected areas being the cortex and the striatum, two regions important for motoric functions (Peters, 2006). On the contrary, in the case of neuropathologic conditions, the observed gray matter loss is mainly attributed to neurodegeneration and is referred to as brain atrophy. SOD2^ako^ mice did not show obvious neuronal death in the brain. However, we detected neuronal soma shrinkage accompanied by DNA damage at the caudoputamen of the striatum. Both of these are signs of an unhealthy and dysfunctional neuronal state and potentially contribute to the observed phenotype. This observation suggests that the observed brain shrinkage resembles most likely an accelerated aging state. However, it is important to note that brain aging and age‐related neurodegenerative diseases share many characteristics and an effort to separate them might be difficult.

Motoric alterations can be attributed to a plethora of factors; thus identifying the etiology can be challenging. The most frequent reasons behind motoric impairment are neuronal damage or death and myelin insults. While we did not observe any neuronal loss in the brain, we noticed a loss of motor neurons in the spinal cord which was accompanied by astrogliosis, microgliosis and denervation of neuromuscular junctions in elder mutants. Importantly, astrogliosis and microgliosis preceded the neuronal loss. Since transcriptome analysis revealed a polarization of SOD2^ako^ astrocytes toward the A1 neurotoxic phenotype, we hypothesize that the reactive astrocytes could initiate or contribute to motor neuron degeneration. Nevertheless, motor neuron loss occurred later in life. This suggests that the initiation of motoric impairment cannot be attributed to spinal motor neuron loss but it may explain the progression to a more severe motoric phenotype.

There is increasing awareness of the importance of astrocytic metabolism and its impact on CNS functions. Strong evidence indicates that astrocytes can adapt their metabolism to support the needs of neurons while they also contribute to myelination by supplying oligodendrocytes with lipids (Camargo et al., 2017; Saab et al., 2013). Further, their metabolic activity is coupled with the intracellular and extracellular ion concentration, directly affecting many of their homeostatic functions. Therefore, slight alterations in their metabolism can have a strong impact on CNS function. Brain hypometabolism is observed physiologically with aging but also in pathologic conditions (Beard et al., 2022; Mulica et al., 2021) while it is still not clear if these metabolic alterations represent cause or consequence in this context. Furthermore, astrocytic dysfunction has been connected to demyelinating disorders like Multiple Sclerosis (Sofroniew & Vinters, 2010), while studies have attempted to associate alterations of astrocytic metabolism with the pathogenesis of the disease (Cambron et al., 2012). Our GSEA revealed reduced transcription of metabolism‐associated genes in the elder SOD2^ako^ astrocytes, indicating a hypometabolic state and potentially, as a result, deregulation of multiple homeostatic functions. Moreover, reduced transcription of lipid metabolism genes suggests decreased lipid supply to the oligodendrocytes, which could explain the myelin impairment and increased g‐ratio observed in the elder mutants. Myelin impairment and loss of myelination are naturally occurring with age (Liu et al., 2017), further supporting our previous accelerated‐aging observations.

In general, astrocytes carry out multiple energy‐demanding functions, thus being highly metabolically active. They depend on oxidative phosphorylation under resting conditions while there is an increase in glycolysis under conditions of increased neuronal activity (Hertz et al., 2007). It has been shown previously that disruption of OxPhos in astrocytes increased glycolysis, most likely as a compensatory mechanism, without any overt effect on the health of the animals (Supplie et al., 2017). Conversely, disruption of OxPhos in astrocytes has a detrimental effect on animals undergoing ischemic stroke, increasing neuronal death in the perilesional area (Fiebig et al., 2019). These data suggest that inhibition of OxPhos alone is not sufficient to induce pathologic conditions in the CNS. However, it affects the homeostatic attributes of the astrocytes under stressful conditions.

Finally, Vicente‐Gutierrez et al recently showed that reduction of mROS in astrocytes leads to metabolic shifts and motoric hypoactivity but has no overall effect on the health and lifespan of the animals (Vicente‐Gutierrez et al., 2019). Specifically, they used an astrocyte‐specific mitochondrial‐tagged catalase mouse model (GFAP‐mCAT), where mROS levels were reduced. Paradoxically, this triggered an elevation in the pentose phosphate pathway (PPP) activity, a pathway usually upregulated under conditions of oxidative stress and critical in the antioxidant mechanism. This elevation however, was attributed to a decrease in the levels of miR‐206, which negatively regulates PPP. Elevated PPP activity led to reduced glycolytic activity, possibly a consequence of increased glucose breakdown by PPP.

In our study, deletion of SOD2 in astrocytes led to a striking drop in mitochondrial respiration. In notion with the aforementioned study, these metabolic alterations are not attributed solely to a direct effect of SOD2 loss on mitochondrial functionality since we also observed a depleted OxPhos RNA signature, which consists mostly of nuclear DNA genes. This suggests a more complex mechanism regulating the activity of OxPhos in SOD2^ako^ mice. In line with the GFAP‐mCAT study where reduction of mROS led to increased PPP activity, ablation of the mitochondrial antioxidant SOD2 induced downregulation of PPP‐associated genes in astrocytes. Conversely though, decreased activity of both OxPhos and PPP did not lead to an increase in glycolysis.

## 4 CONCLUSIONS

Oxidative stress is a crucial factor in the initiation and acceleration of aging. Our study provides evidence that SOD2 function in astrocytes is a major factor affecting CNS and organismal aging. We propose that redox imbalance at astrocytes dampens multiple metabolic processes, rendering them unable to fulfill their homeostatic functions, while also pushing them toward a highly inflammatory neurotoxic A1 phenotype. This A1 phenotype is able to exert non‐cell‐autonomous detrimental effects on CNS function, which ultimately leads to severe motor dysfunction and premature death. Overall, our data consolidate the significance of physiological ROS levels maintenance in astrocytes as a prerequisite for healthy brain aging.

## 5 MATERIALS AND METHODS

### 5.1 Mouse line generation

The mouse model was generated by crossing mice expressing Cre recombinase under the hGFAP promoter (hGFAP‐Cre^+/−^) (Zhuo et al., 2001) with mice carrying one floxed SOD2 (SOD2^fl/+^) allele (Strassburger et al., 2005) to generate hGFAP‐Cre^+/−^;SOD2^fl/+^ mice. In a second breeding step, hGFAP‐Cre^+/−^ SOD2^fl/+^ mice were crossed with SOD2^fl/fl^ mice to generate astrocyte‐specific SOD2‐deficient (hGFAP‐Cre^+/−^; SOD2^fl/fl^) mice. The mice (all C57BL/6) were kept at the animal facility of the University of Ulm (12‐h light–dark cycle) and had access to food and water ad libitum. Mice of either sex were used in this study. Littermates carrying various genotypes but not expressing Cre recombinase were used as controls and were designated as WT mice. The study was conducted according to the guidelines of the Declaration of Helsinki. All experiments were performed in compliance with institutional guidelines (Tierforschungszentrum TFZ, Ulm University) and German Animal Protection Act and approved by the Regierungspräsidium Tübingen (Tübingen, Germany).

### 5.2 Genomic DNA analysis

Genomic DNA was prepared from tissue (cerebrum, cerebellum, spinal cord, tail tips) or cells using the commercially available KAPA Mouse Genotyping Kit (Roche, #KK5621). Recombination and deletion of SOD2 floxed site was determined by PCR using purified Genomic DNA. In the case of SOD2, presence of an ~850 bp band signifies the flox allele, whereas a band at 786 bp denotes the wild‐type allele and a band at ~200 bp represents the recombined allele. The primer sequences are provided in Table S1.

### 5.3 Protein isolation and Western blot

Tissue (cerebrum, cerebellum, spinal cord) was homogenized in RIPA buffer (Pierce) containing protease and phosphatase inhibitors (Pierce) using TissueLyser II (Qiagen). Cells extracted via an immunomagnetic approach were snap‐frozen in liquid nitrogen and stored at −80°C. The cells were resuspended in buffer containing 4% sodium dodecyl sulfate (SDS), 100 mM Tris–HCl, protease and phosphatase inhibitors. The protein content in the tissue or cell lysate was determined by the Pierce™ BCA Protein Assay Kit (Thermo Fisher Scientific, #23227). Western blots were performed according to standard protocols. An antibody list is provided in Table S2.

### 5.4 Health and motor‐behavioral assessment

#### 5.4.1 Body weight, symptoms, and humane endpoint assessment

Body weight (BW) was measured every 5 weeks from the age of 10 weeks onward. Starting from the age of 30 weeks, daily monitoring of symptoms was performed: impaired mobility, tremors, abnormal gait, kyphosis, bradykinesia, and paresis were annotated. Extra wet food was provided if needed. The humane endpoint was set at paresis onset or when the mice weighted less than 80% of the corresponding wild‐type littermates. For the motor‐behavioral analysis, only male mice were used.

#### 5.4.2 Rotarod

To assess motor coordination and strength, a rotarod apparatus (Ugo Basile, #47600) was used. To familiarize themselves with the apparatus, mice underwent daily training, for four days, where three trials with an accelerating speed from 5 rpm to 40 rpm (doubling speed daily) were performed. For the next 2 days, 3 trials daily, totaling 6, were performed to evaluate the motor coordination of mice. Every trial lasted a maximum of 300 s. The latency to fall from the rotating rod was recorded in each trial. If a mouse was passively rotating on the rod (i.e., clinging) the number of passive rotations was counted (Marazziti et al., 2013). Data were expressed as mean latency to fall minus 5 s for each passive rotation. If the latency minus the penalty was lower than the moment of the first passive rotation, then the moment of the first passive rotation was used for the calculation of the mean.

#### 5.4.3 Open field

To assess activity and exploratory behaviour, mice underwent the open field test. Mice were individually placed in the center of a cubic arena (open field box 36 × 36 × 18 cm) made of grey Plexiglas and allowed to habituate for 10 min (novel field test). Directly after the habituation, the mice were placed again in the exact same arena and allowed to explore freely for 10 more minutes (familiar field test). After each trial, the arena was wiped with 70% ethanol and let dry for 3 min to avoid odorant cues from other mice or the researcher. After data acquisition, the total distance moved, average velocity and corner‐to‐center ratio for each mouse were calculated.

#### 5.4.4 Beam walk

To assess motor coordination and balance, we evaluated the ability of mice to traverse a narrow beam of 1 cm diameter to reach an enclosed safety platform. Beams were horizontal and 20 cm above the table. Mice were trained to traverse the beam for 2 days. The next day, 3 trials were performed, where the success or failure of each mouse to traverse the beam was recorded.

#### 5.4.5 Grip strength

Mice were subjected to quantitative grip‐strength assessment using a commercial apparatus (BioSeb, #BIO‐GS3). The mouse was held gently by the base of its tail over the top of the grid enabling both forelimbs and hindlimbs to grip the grid. With its torso in a horizontal position, the mouse was pulled back steadily until the grip was released down the complete length of the grid. The peak tension was measured in grams. The mean of two trials was taken as an index of forelimb and hindlimb grip strength. Mice were given an inter‐trial interval of about 60 s.

### 5.5 Brain size quantification

For brain size quantification, fresh brains were dissected and directly placed on a ruler and high‐resolution pictures were acquired. For brain length, a line crossing the cerebrum and cerebellum was drawn and quantified. For cerebrum and cerebellum width, the largest vertical line to the brain length line was selected and measured. Quantification of the size was performed with ImageJ (Rasband, W.S., ImageJ, U. S. National Institutes of Health).

### 5.6 Histology and immunofluorescence

For paraffin sections, brains were dissected, fixed with 4% paraformaldehyde (overnight at 4°C) and then embedded in paraffin using a Tissue Processor (Microm STP 120, Thermo Fisher Scientific). 7 μm thick coronal sections were used for routine histology and other immunostainings. Heat‐mediated antigen retrieval was performed with 10 mM sodium citrate buffer (pH 6). Sections were washed in PBS, blocked with 5% bovine serum albumin and 1% Fc block for 1 h at room temperature, and then incubated with primary antibodies overnight at 4°C.

For free‐floating (brain and spinal cord) or cryosections (gastrocnemius), animals were anesthetized with an intraperitoneal injection of a drug cocktail containing ketamine (100 mg/kg) and xylazine (10 mg/kg) diluted in a 0.9% NaCl solution. After anesthesia, transcardial perfusion was performed, pumping 25 mL of PBS, followed by 50 mL of 4% PFA in PBS as a fixative. Later, brains, spinal cords and gastrocnemius muscles were extracted and embedded in 4% PFA for 2 h for post‐fixation before they were incubated in 30% saccharose in PBS overnight at 4°C for cryoprotection. Free‐floating (20 μm for brains, 40 μm for spinal cords thick) coronal sections were prepared and stored in PBS at 4°C for 7 days or in 30% ethylene glycol, 20% glycerol in 1×PBS at −20°C for longer periods, while 30 μm cryosections for gastrocnemius muscles were kept at −80°C. For staining, slices were blocked and permeabilized in PBS, 0.5% Triton‐X™, 10% goat serum and 1% Fc block for 1 h. After washing with PBS, sections were incubated with primary antibodies overnight at 4°C.

For cell cultures, primary astrocytes were seeded on laminin‐coated (20 μg/mL; #10267092; Fisher Scientific) glass coverslips (Carl Roth GmbH) and were incubated in conventional culture conditions for 5 days and further used for immunofluorescence analyses.

In either paraffin sections, free‐floating sections or cryosections, slices were washed with PBS and incubated with secondary antibodies for 1 h at room temperature on the following day after incubation with the primary antibody. DAPI was applied for nuclear counterstaining. An antibody list is provided in Table S3. Analysis of the images was performed with ImageJ software.

For caudoputamen mediolateral size quantification, caudoputamen slices from same area were selected. A line vertical to the lateral ventricle, starting from the middle of the lateral ventricle and extending to the corpus callosum was drawn and measured.

For size quantification of reactive astrocytes and neurons, brain slices were stained with antibodies against GFAP and NeuN respectively, and the corresponding area covered by each cell was quantified.

For the quantification of motor neurons from the lumbar region of the spinal cord, the average number of VACHT^+^ cells between the left and the right side of every section was calculated.

For quantification of denervated neuromuscular junctions, junctions without any trace of synaptophysin colocalization with a‐Bungarotoxin were considered as such.

### 5.7 Image acquisition

Images were acquired with an All‐in‐one fluorescence microscope BZ‐X810 from Keyence equipped with filters for DAPI, FITC, DsRed, Texas Red and Cy5. Software images were acquired with the BZ‐X800 software. Images of stained sections were captured with equal exposure times per channel and the same graphical pre‐processing within one experiment.

### 5.8 Immunomagnetic purification of astrocytes from the adult brain

Mouse adult brain tissue was dissociated with an adult mouse brain dissociation kit (Miltenyi Biotec, #130‐107‐677). Dissociated cells, after removal of debris and red blood cells, were separated magnetically with an astrocyte‐specific anti‐ACSA‐2 Microbead Kit (Miltenyi Biotec, #130‐097‐678). We confirmed the identity of the isolated fractions by flow cytometry against astrocytic‐specific marker ACSA‐2 (Miltenyi Biotec, #130‐117‐535). The flow cytometry was performed in BD FACS CANTO II flow cytometer (BD Biosciences). The software used for data acquisition was BD FACSDIVA and the software used for data analysis was FlowJo_v10.7.2.

### 5.9 RNA sequencing

Astrocytes were purified from brains of elder moribund SOD2^ako^ mice and their age‐matched littermates via an immunomagnetic approach. Two brains were pooled for every sample. Subsequently, RNA was extracted using the RNeasy mini kit (Qiagen, #74104). Integrity of RNA was checked with Agilent 2100 bioanalyzer (Agilent). cDNA library construction and sequencing were performed by the Novogene Co., Ltd. (Novogene) with Illumina NovaSeq 6000 Sequencing System. Mapping of reads to the reference genome mm10 was done with STAR v2.6.1d (Dobin et al., 2013). Differentially expressed genes were calculated and discovered with the NovoSmart software provided by Novogene Co., Ltd. Parameters for differential genes analysis were *p* < 0.05, *q* < 0.1 and fold change > 1.5. Additional analysis was performed with GSEA (version 4.2.1) software (Subramanian et al., 2005). For the GSEA graphs, the false discovery rate (FDR) and the normalized enrichment score (NES) are also displayed. For the generation of the A1 reactive astrocytes geneset, 24 genes based on recent classification were selected (Boisvert et al., 2018). The list is provided in Table S4. To generate heat maps and Principal Component Analysis (PCA), Rstudio and the libraries “gplots”, “ggplot2” and “rgl” were used. Four biological replicates per genotype were analyzed.

### 5.10 Primary cell cultures, cellular respiration and extracellular acidification

Cell suspensions of acutely isolated astrocytes by immunomagnetic purification were seeded in 12‐well plates (Corning, #351143) at a density of 130,000 cells/well in DMEM with glucose, GlutaMAX(TM) and pyruvate (Thermo Fisher Scientific, #31966) supplemented with 10% fetal bovine serum and 1% penicillin–streptomycin. The medium was exchanged every second day. On day 10, the cells were transferred in XF96 V3 PS cell culture Microplates (Agilent Technologies) coated with 0.01% Poly‐d‐Lysin (Thermo Fisher Scientific) at a density of 40,000 cells/well in the same culture medium and the Seahorse assay took place 5 days later. On the day of measurement, the cells were washed with XF assay medium (Agilent Technologies) containing 1 mM sodium pyruvate and 2 mM glutamine and incubated in fresh XF medium for 1 h under CO_2_‐free conditions. Thereafter, oxygen consumption and extracellular acidification rates (OCR and ECAR) were measured simultaneously using a Seahorse XFe96 Flux Analyzer (Agilent Technologies). The following injections and final concentrations were used: glucose (1 μM) oligomycin (2 μM), FCCP (2 μM) and antimycin A (0.5 μM)/rotenone (0.5 μM). Data were normalized to total DNA per well as a surrogate for cell number per well.

### 5.11 Electron microscopy

Animals were anesthetized with an intraperitoneal injection of a drug cocktail containing ketamine (100 mg/kg) and xylazine (10 mg/kg) diluted in a 0.9% NaCl solution. After anesthesia, mice were sacrificed with decapitation. Brains were quickly dissected and fixed in 0.1% Glutaraldehyde, 4% PFA, 2% Sucrose in 0.1 M Sorensen Phosphate Buffer for 24 h. The area of interest, caudoputamen, was dissected and fixed again overnight in 2.5% Glutaraldehyde, 0.1% Sucrose in 0.1 M Sorensen Phosphate Buffer. Samples were then washed in PBS and post‐fixed in 2% osmium tetroxide in PBS. After dehydrating the samples in a graded series of isopropanol, they were blockstained in 2% uranyl acetate in ethanol and embedded in Epon. Semi‐thin (500 nm) sections were stained with toluidine blue and analyzed using light microscopy to find the area of interest. Ultra‐thin sections (80 nm) were cut on a microtome using a diamond knife (Leica EM UC7) and collected on carbon‐coated formwar films on 200 mesh copper grids (Plano) contrasted with 0.3% lead citrate for 1 min and imaged using a TEM 1400 (Jeol). For the experiments, only axons localized in axon fiber bundles passing through the caudoputamen were chosen. For the quantification of non‐myelinated axons, only axons with caliber over 200 nm were chosen. Analysis of the images was performed with ImageJ software.

### 5.12 Statistics

Statistical analyses were performed with Graphpad Prism v.8.4.3. Diagrams show arithmetic means and standard deviations (SDs). Two‐tailed student's *t*‐test with Welch's correction was used for the comparison of 2 age‐matched groups (*p < 0.05, **p < 0.01, ***p < 0.001, ****p < 0.0001). For survival analysis, each group was examined by Kaplan–Meier survival estimators and the survival outcomes were compared using log‐rank test. For body weight comparison, two‐way analysis of variance (ANOVA) with Bonferroni correction and multiple comparisons between the age‐matched groups was used for every sex (*p < 0.05). One‐way ANOVA with Bonferroni correction and multiple comparisons was used for the comparison of more than 2 groups (*p < 0.05, **p < 0.01, ***p < 0.001).

## AUTHOR CONTRIBUTIONS

**Konstantinos Tsesmelis:** Methodology; Software; Validation; Formal analysis; Investigation; Writing – original draft; Visualization; Project administration. **Gandhari Maity‐Kumar:** Conceptualization; Methodology; Writing – review & editing; Funding acquisition. **Dana Croner:** Investigation. **Jasmin Sprissler:** Investigation. **Miltiadis Tsesmelis:** Investigation; Writing – review & editing. **Tabea Hein:** Investigation. **Bernd Baumann:** Writing – review & editing; Supervision. **Thomas Wirth:** Conceptualization; Methodology; Resources; Data curation; Writing – review & editing; Supervision; Project administration; Funding acquisition.

## CONFLICT OF INTEREST STATEMENT

The authors declare no conflict of interest.

## Supporting information


Figure S1

Figure S2

Figure S3

Figure S4

Table S1

Table S2

Table S3

Table S4

Video S1

Appendix S1

## Data Availability

All data generated in this study are included in this published article. Further RNA‐Sequencing data will be made available to the public prior to publishing. All custom scripts are available from the corresponding authors upon reasonable request.
