# Supplementary figures and images for: Accelerated aging in mice with astrocytic redox imbalance as a consequence of SOD2 deletion
Source: Aging Cell. 2023 Aug 23;22(9):e13911. doi: 10.1111/acel.13911 (PMC10497807; doi:10.1111/acel.13911)

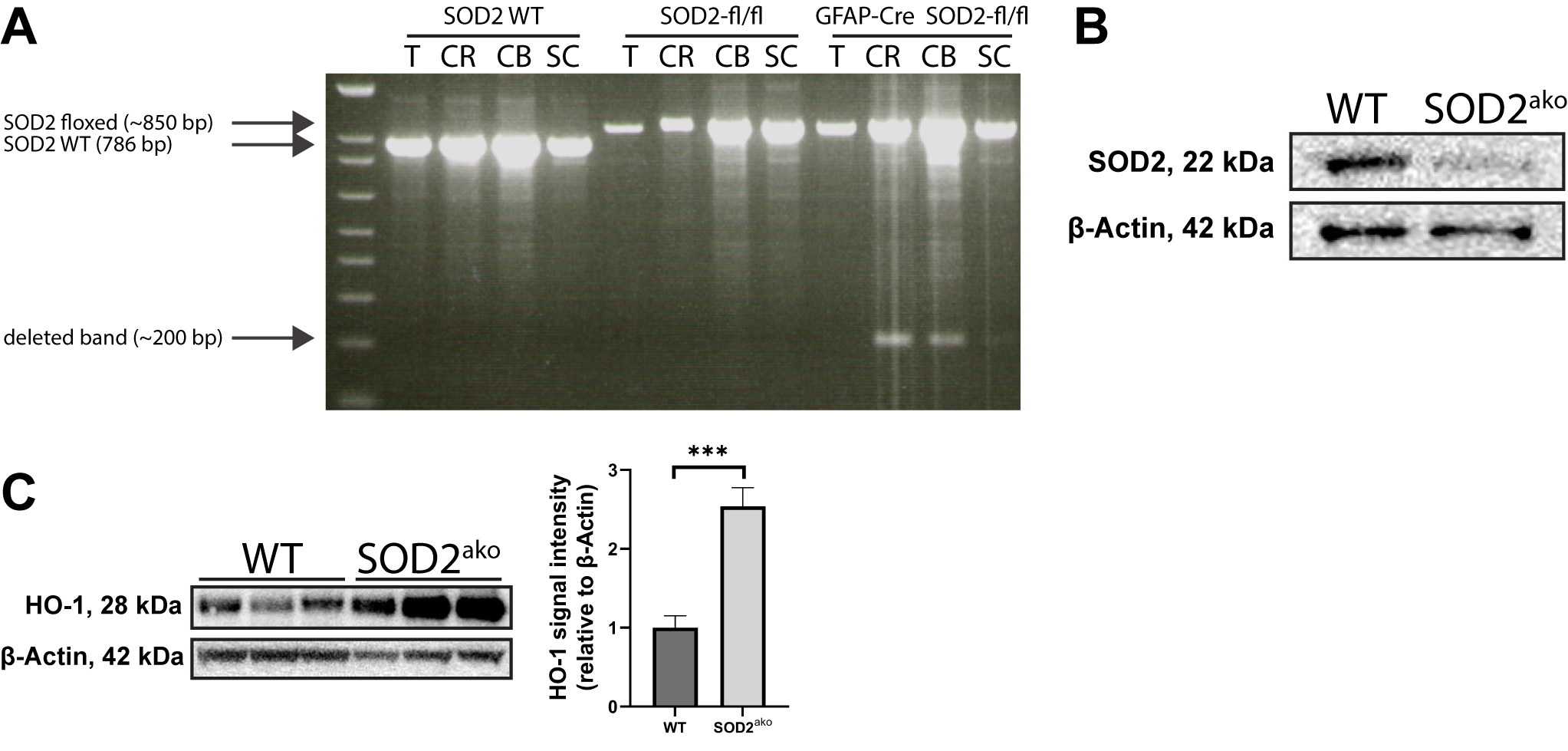

Supplement: Supplementary file 1 — Figure S1 [file ACEL-22-e13911-s009.tif]

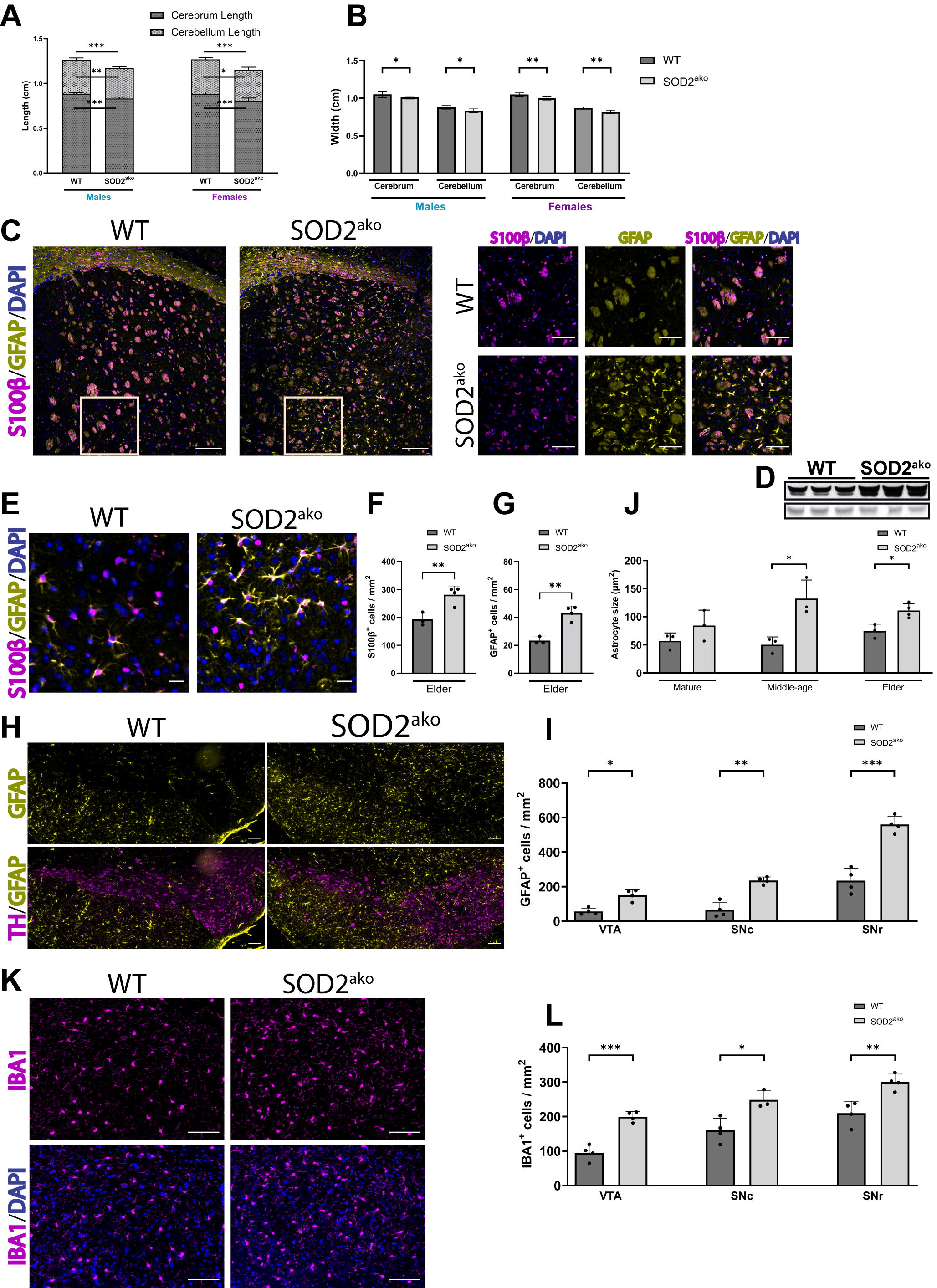

Supplement: Supplementary file 2 — Figure S2 [file ACEL-22-e13911-s008.tif]

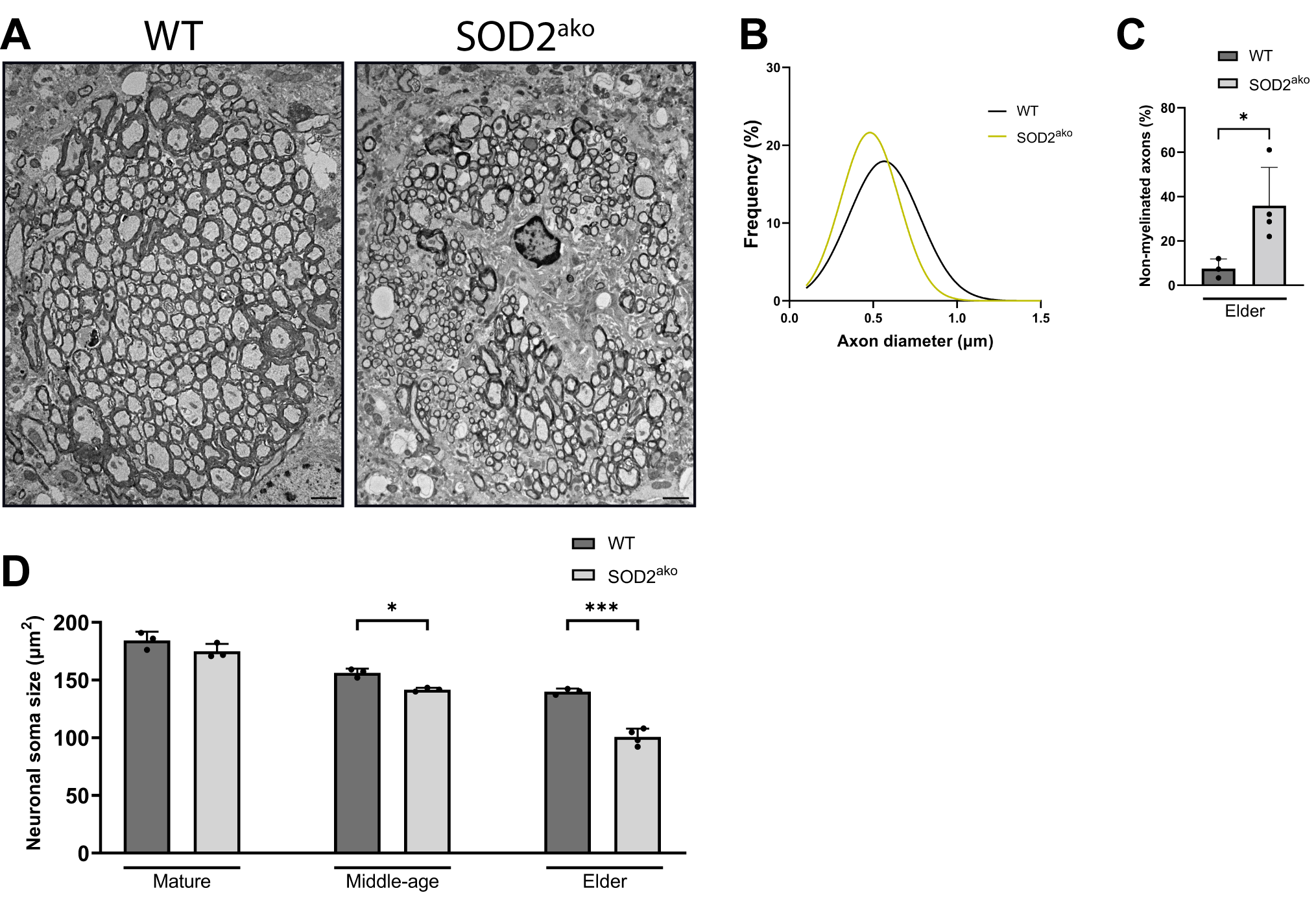

Supplement: Supplementary file 3 — Figure S3 [file ACEL-22-e13911-s004.tif]

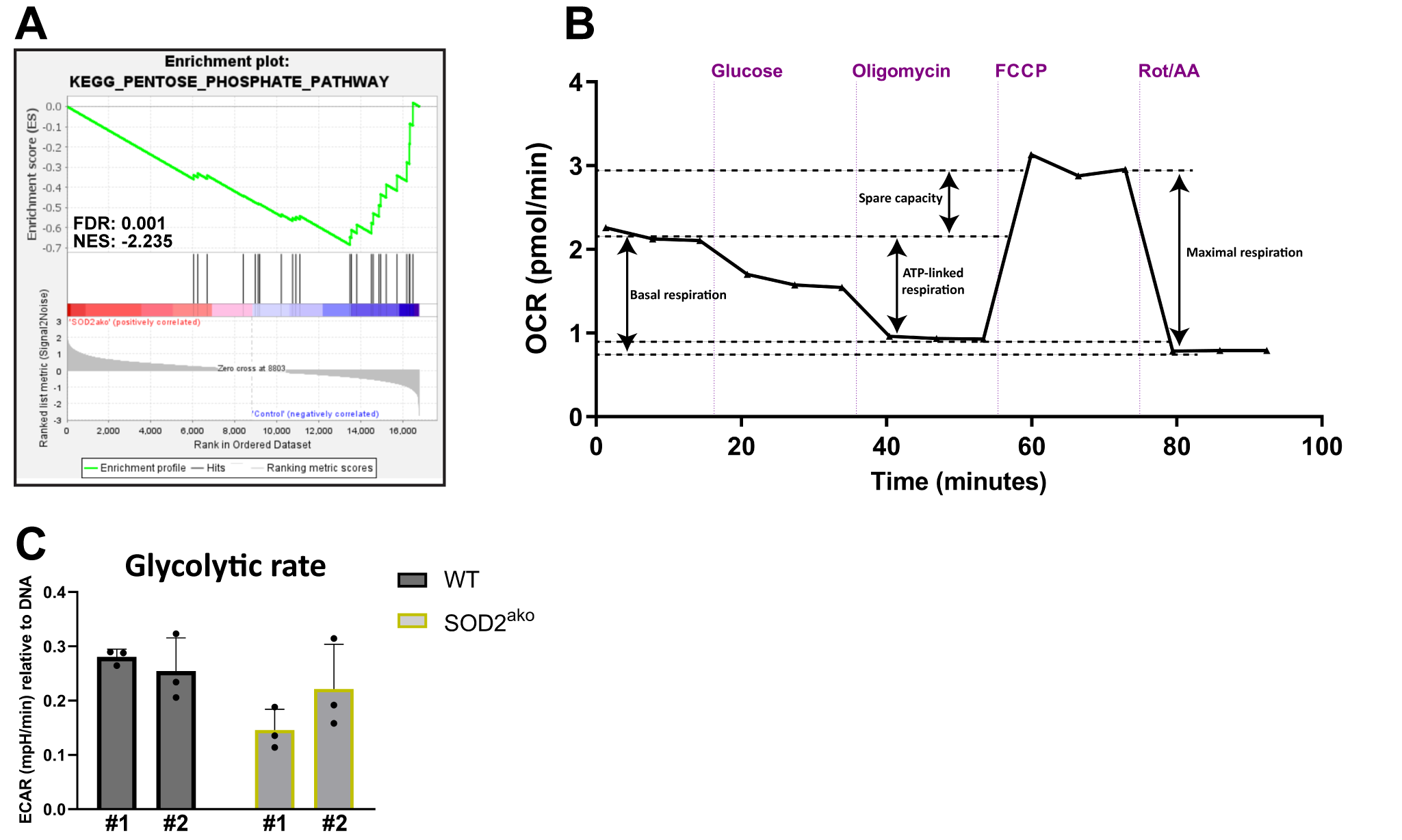

Supplement: Supplementary file 4 — Figure S4 [file ACEL-22-e13911-s005.tif]
