# Supplementary material for: Accelerated aging in mice with astrocytic redox imbalance as a consequence of SOD2 deletion
Source: Aging Cell. 2023 Aug 23;22(9):e13911. doi: 10.1111/acel.13911 (PMC10497807; doi:10.1111/acel.13911)
Supplement: Supplementary file 10 — Appendix S1 [file ACEL-22-e13911-s010.docx]

**Video 1:** SOD2^ako^ mice displayed psychotic moments.

In this 25-second interval of the captured psychotic moment, the SOD2^ako^ mouse reached a 5-fold higher velocity than its average velocity and a 3-fold higher velocity than the average of the age-matched WT littermate. Left: SOD2^ako^, Right: WT.

**Figure S1:** Model validation.

**(A)** PCR analysis from tail, cerebral, cerebellar and spinal cord genomic DNA. T: tail, CR: Cerebrum, CB: Cerebellum, SC: Spinal cord. **(B)** Western blot analysis of primary astrocytes against SOD2. β-Actin was used as a loading control. **(C)** Western blot analysis of cerebral protein lysates against HO-1. β-Actin was used as a loading control. Data shown as mean ± SD; n = 3 mice/group; student’s t-test: *** p < 0.001.

**Figure S2:** SOD2^ako^ mice develop astrogliosis and microgliosis in motoric brain regions.

**(A),** **(B)** Quantification of brain size (length and width respectively) of elder mice. Data shown as mean ± SD; n_WT-males_ = 9 mice, n_SOD2ako-males_ = 5 mice, n_WT-females_ = 9 mice, n_SOD2ako-females_ = 6 mice; student’s t-test: * p < 0.05, ** p < 0.01. **(C)** Immunostaining against GFAP at the caudoputamen of mature mice (anti-S100β: red; anti-GFAP: green, DAPI: blue). Full image scale bar: 200 μm, inset scale bar: 100 μm. **(D)** Western blot of middle-age cerebellar protein lysates against GFAP. β-Actin was used as a loading control. **(E)** Immunostaining against GFAP and S100β at the motor cortex of elder mice (anti-S100B: red; anti-GFAP: green, DAPI: blue). Full image scale bar: 20 μm. **(F)** Quantification of S100β^+^ cells at the motor cortex of elder mice. Data shown as mean ± SD; n_WT_ = 3 mice, n_SOD2ako_ = 4 mice; student’s t-test: ** p < 0.01. **(G)** Quantification of GFAP^+^ cells at the motor cortex of elder mice. Data shown as mean ± SD; n_WT_ = 3 mice, n_SOD2ako_ = 4 mice; student’s t-test: ** p < 0.01. **(H)** Immunostaining against GFAP at the substantia nigra of elder mice (anti-TH: red; anti-GFAP: green, DAPI: blue). Full image scale bar: 100 μm. **(I)** Quantification of GFAP^+^ cells at the Ventral Tegmental Area (VTA), Substantia nigra pars compacta (SNc), Substantia nigra pars reticulata (SNr). Data shown as mean ± SD; n = 4 mice/group; student’s t-test: * p < 0.05, ** p < 0.01. **(J)** Quantification of GFAP^+^ cell size at the caudoputamen throughout aging. Data shown as mean ± SD; n = 3-4 mice/group; student’s t-test: * p < 0.05. **(K)** Immunostaining against IBA1 at the substantia nigra of elder mice (anti-IBA1: red, DAPI: blue). Full image scale bar: 20 μm. **(L)** Quantification of IBA1^+^ cells at the VTA, SNc, SNr of elder mice. Data shown as mean ± SD; n = 4 mice/group; student’s t-test: * p < 0.05, ** p < 0.01, *** p < 0.001.

**Figure S3:** Elder SOD2^ako^ mice have shrunk neurons and an increased number of non-myelinated axons.

**(A)** Electron microscopy analysis revealed the presence of some irregular formation at axon fiber bundles of the caudoputamen of elder SOD2^ako^ mice. A cell, probably phagocytosing microglia, is present in this SOD2^ako^ axon fiber. Scale bar: 2 μm. **(B)** Gaussian distribution of axonal diameter at the caudoputamen of elder mice. n_WT_ = 784 axons, n_SOD2ako_ = 621 axons. **(C)** Quantification of non-myelinated axons at the caudoputamen of elder mice. Data shown as mean ± SD; n_WT_ = 3 mice, n_SOD2ako_ = 4 mice; student’s t-test: * p < 0.05. **(D)** Quantification of neuronal soma size at the caudoputamen throughout aging. Data shown as mean ± SD; n = 3-4 mice/group; student’s t-test: * p < 0.05, *** p < 0.001.

**Figure S4:** SOD2^ako^ astrocytes are in a hypometabolic state.

**(A)** GSEA of the pentose phosphate pathway from astrocytes of elder mice. n = 4 mice/group. KEGG Pentose Phosphate Pathway: FDR = 0.001, NES = -2.235. **(B)** Schematic approach for the calculation of basal respiration, ATP-linked respiration, maximal respiration and spare capacity based on the oxygen consumption rate results from a Seahorse assay. **(C)** Quantification of the glycolytic rate from a Seahorse assay. Data shown as mean ± SD; n = 3/group; 1-way ANOVA with Bonferroni correction and multiple comparisons between the WT and SOD2^ako^ groups.
